# Description of a new species of *Surazomus* (Arachnida: Schizomida), with comments on homology of male flagellum and mating march anchorage in the genus

**DOI:** 10.1371/journal.pone.0213268

**Published:** 2019-03-20

**Authors:** Gustavo R. S. Ruiz, Roberta M. Valente

**Affiliations:** Instituto de Ciências Biológicas, Universidade Federal do Pará, Belém, Pará, Brazil; University of Georgia, UNITED STATES

## Abstract

*Surazomus saturninoae* sp. nov. is described from eastern Amazon. The male has a pentagonal flagellum, similar to those of three other species in the genus. These four species are herein gathered as the *arboreus*-group of *Surazomus*. We present a brief synopsis of chaetotaxy description in hubbardiines and several homology proposals for the flagellum of the species in the *arboreus*-group: the posterior lobes may be homologous to the lateral lobes of hubbardiine species with trilobed flagella; the setal brush with 4–5 setae on the posterior lobe may be composed of one Dl2 seta and enlarged lobular microsetae; the single, median posterior coupling pocket may be homologous to the pair of posterior pockets seen in other hubbardiines; the single, median anterior coupling pocket may be homologous to the pair of pockets on the anterior border of the flagellum seen in other hubbardiines. Based on the morphology of these pockets and the chelicerae within *Surazomus*, we discuss the anchoring mechanism during the mating march.

## Introduction

Schizomids are small arachnids that display short-range endemism [1,2,3] and generally occur in humid tropical and sub-tropical forests, mainly in leaf litter, caves, tree bark or under stones [4]. The extant diversity of about 300 species of Schizomida is divided into the families Protoschizomidae and Hubbardiidae, the last with two subfamilies: Hubbardiinae and Megaschizominae [5]. They are all known as short-tailed whip scorpions, due to the presence of a short flagellum in males and females. The flagellum of the male is highly modified and held by the chelicerae of the female during copulation [6,7], in a phase called as *Paarungsmarsch* (= mating march) [7], when she is dragged forward onto the spermatophore. The anchoring mechanism is still poorly understood, but the male flagellum is certainly sexually selected, as it has diverse forms throughout the order, rendering them useful in systematics [8,9,10]. Albeit diverse, the flagellum of schizomids bears multiple setae that are mostly constant within species and are present across the major taxonomic groups, being largely used in species descriptions and hypotheses of setal homology since the 1990s [11,9,12,13,14]. Recently, Monjaraz-Ruedas *et al*. [15] compared the setae of Protoschizomidae, Hubbardiinae and Megaschizominae to reevaluate homologies and reconstruct the phylogeny of the order. Even with the achievements of that study, the homology of some setae is still debatable, such as the lateral pair over the flagellar stalk of some male hubbardiines [16].

Presently *Surazomus* Reddell & Cokendolpher, 1995 (Hubbardiidae: Hubbardiinae) includes 24 species distributed through Central and South America: seven species from Costa Rica, seven from the Andean foothills and ten from the Amazon region [3,13,17,18]. Within *Surazomus* we find different types of male flagella: some species have a rounded flagellum, such as *Surazomus chavin* Pinto-da-Rocha, 1996 and *Surazomus uarini* Santos & Pinto-da-Rocha, 2009 (see Armas [3]: figs 13A and 19A); many have a trilobed flagellum bearing a pair of membranous dorso-submedian eminences, with the median portion of the flagellum laterally widened (e.g. *Surazomus kitu* Villarreal, Miranda & Giupponi, 2016; see Villarreal *et al*. [13]: fig 4A); and a few have a pentagonal flagellum with posterior lobes, such as *Surazomus arboreus* Cokendolpher & Reddell, 2000 (see Armas [3]: fig 11B). The different shapes of male flagellum within *Surazomus* are still poorly understood in terms of evolution, as well as the flagellar chaetotaxy [13]. Despite having never been used in phylogenetic reconstructions of *Surazomus*, the male flagellum and chaetotaxy may define monophyletic groups within the genus. However, the homology between portions of different flagellar types and their setae still needs further comparative studies.

Herein we describe a new species of *Surazomus* from eastern Amazon with a highly modified pentagonal male flagellum. Its unusual morphology provided us insights about changes that may have occurred in the male flagellum of a group of species, allowing us to propose new homology hypotheses between portions and setae of the flagellum within the genus.

## Material and methods

The type specimen is deposited in the Museu Paraense Emílio Goeldi, Belém, Brazil (MPEG, A.B. Bonaldo). Multifocus colour photographs were taken with a Leica DFC420 digital camera attached to a Leica M205A microscope, and stacked with Leica application suite version 3.4.1, which was also used to take the measurements, expressed in millimeters. Drawings were prepared with a Leica DM 1000 microscope and a drawing tube. For illustration of chelicera setation and pedipalp setation, the whole structures were dissected and immersed in clove oil.

Nomenclature follows Reddell & Cokendolpher [10] for appendage articles, Villarreal *et al*. [12,13] for opisthosomal setation, and Lawrence [19], modified by Villarreal *et al*. [13], for cheliceral setation. Pedipalp setation follows Monjaraz-Ruedas & Francke [20,21], including the following new abbreviations: MS: mesal spur; PAP: patellar apophysis; pTS: prolateral tarsal spur; rTS: retrolateral tarsal spur. The nomenclature of flagellar setation follows Monjaraz-Ruedas *et al*. [15]. To compile knowledge on names of flagellar setae through time and compare them with our proposal, we used the table given by Moreno-González *et al*. [14], with a few changes, to keep homologous setae in the same lines.

To discuss setal homology and transformations in male flagellum within *Surazomus*, we compared *S*. *kitu* as a representative of the species with the typical trilobed flagellum (and rounded flagellum, which have similar chaetotaxy), the unusual-shaped “trilobed” flagellum of *S*. *sturmi*, which has posterior lobes in the adult, but is trilobed in the juvenile, and our new species to represent the species with pentagonal male flagellum. The male flagellum of *S*. *kitu* was redrawn in dorsal and lateral views based on Villarreal *et al*. ([13]: figs 4A and 4C). For *S*. *sturmi*, the dorsal view of the flagellum of the juvenile was based on Kraus ([22]: fig 9), while the dorsal view of the adult male flagellum was based on Armas ([3]: fig 18A), and the lateral view was based on Kraus ([22]: fig 8), with seta modifications to match the photograph given by Sturm ([7]: fig 22A). Homologies were established according to the overall shape of the flagella and relative position of setal insertions on the flagellar bodies. Based on the inferred transformations in proportions of homologous areas shown in that illustration, we were able to trace the homologies of the different setae present on the pentagonal flagellum of our new species with those present on the trilobed flagellum of *S*. *kitu*. That species was included in a recent review about flagellar homology and its setae are well-known and understood [13]. With the setal homologies between *S*. *kitu* and our new species established, we aimed to compare the setae of our species with those of the other species that have pentagonal flagella, namely *S*. *arboreus*, *Surazomus manaus* Cokendolpher & Reddell, 2000, and *Surazomus paitit* Bonaldo & Pinto-da-Rocha, 2007.

**Fig 1:**
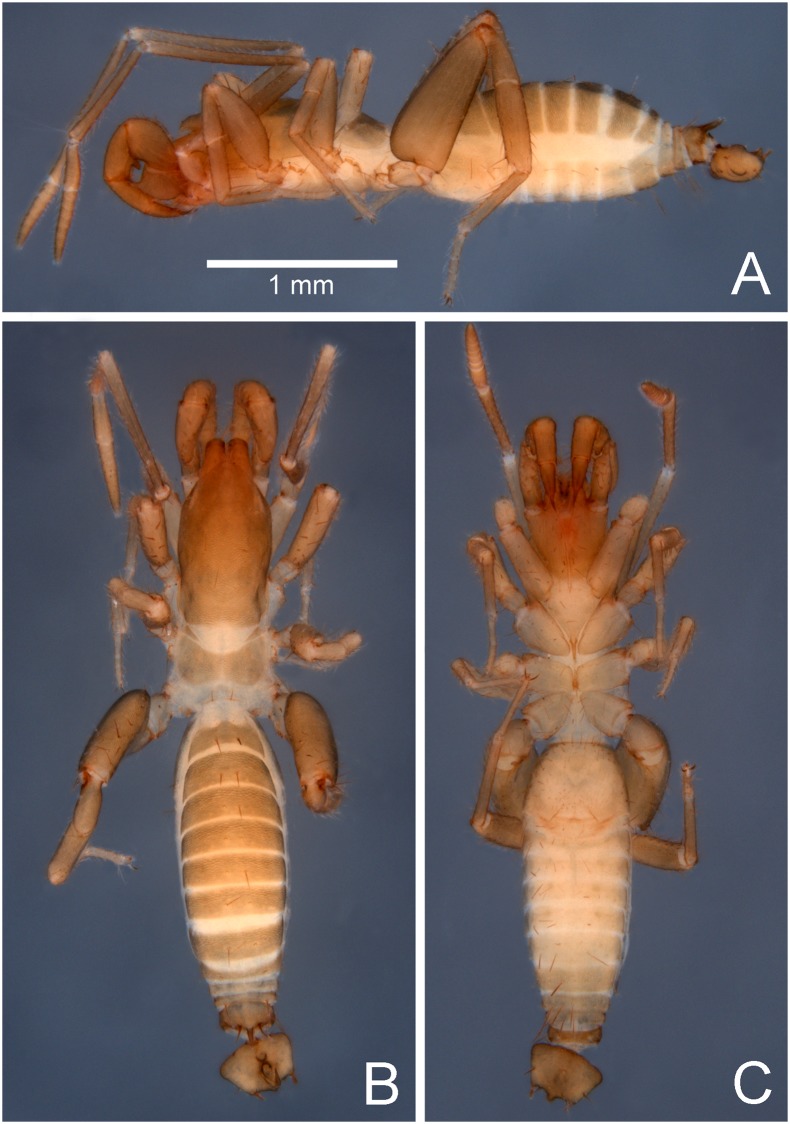
Male holotype of *Surazomus saturninoae* sp. nov. (A) Lateral view. (B) Dorsal view. (C) Ventral view.

Depressions present on the dorsum and venter of the male flagellum are herein interpreted as coupling pockets, as suggested by Sturm [7]. We also compared our new species with the recently described *Surazomus algodoal* Ruiz & Valente, 2017 and *Surazomus rafaeli* Salvatierra, 2018, in order to explain the proposals of homology for setae and coupling pockets of the male flagellum in the genus. Dorsal views of the flagellum of these two species were based on Ruiz & Valente ([17]: fig 11) and Salvatierra ([18]: fig 10), respectively, while the dorsolateral views are free-hand reconstructions based on these and on Ruiz & Valente ([17]: fig 13) and Salvatierra ([18]: fig 8). To elucidate our proposal of homologies for the coupling pockets, we prepared an illustration showing the position of a couple in mating march, based on Sturm ([7]: figs 1 and 21), showing our hypotheses for the anchorage of the female chelicerae onto the male flagellum. We used the male chelicera of the new species to demonstrate a generalized hubbardiine chelicera, and the illustrations cited above [17,18] to show the different character states within the genus. The coupling pockets present on the male flagellum are herein named as “anterior coupling pocket” (AP) and “posterior coupling pocket” (PP).

### Nomenclatural acts

The electronic edition of this article conforms to the requirements of the amended International Code of Zoological Nomenclature, and hence the new name contained herein is available under that Code from the electronic edition of this article. This published work and the nomenclatural acts it contains have been registered in ZooBank, the online registration system for the ICZN. The ZooBank LSIDs (Life Science Identifiers) can be resolved and the associated information viewed through any standard web browser by appending the LSID to the prefix “http://zoobank.org/”. The LSID for this publication is: **urn:lsid:zoobank.org:pub: 55A98F85-A7D8-47B4-B0BA-66374888EB09**. The electronic edition of this work was published in a journal with an ISSN, and has been archived and is available from the following digital repositories: PubMed Central, LOCKSS.

## Results and discussion

### Taxonomy

**Table:** 

**Family Hubbardiidae Cook, 1899**
**Subfamily Hubbardiinae Cook, 1899**
**Genus *Surazomus* Reddell & Cokendolpher, 1995**

#### The arboreus-group of Surazomus

Within *Surazomus*, four species share features believed to be apomorphic and compose a species group, easily distinguished from the remaining species of the genus and traditionally recognized, but unnamed [23,24,13]. To ease the discussion below, we herein propose the use of the informal name “the *arboreus*-group of *Surazomus*” to refer to that group.

**List of species of the *arboreus*-group**: *Surazomus arboreus* Cokendolpher & Reddell, 2000; *Surazomus manaus* Cokendolpher & Reddell, 2000; *Surazomus paitit* Bonaldo & Pinto-da-Rocha, 2007; and *Surazomus saturninoae* **sp. nov**.

**Fig 2:**
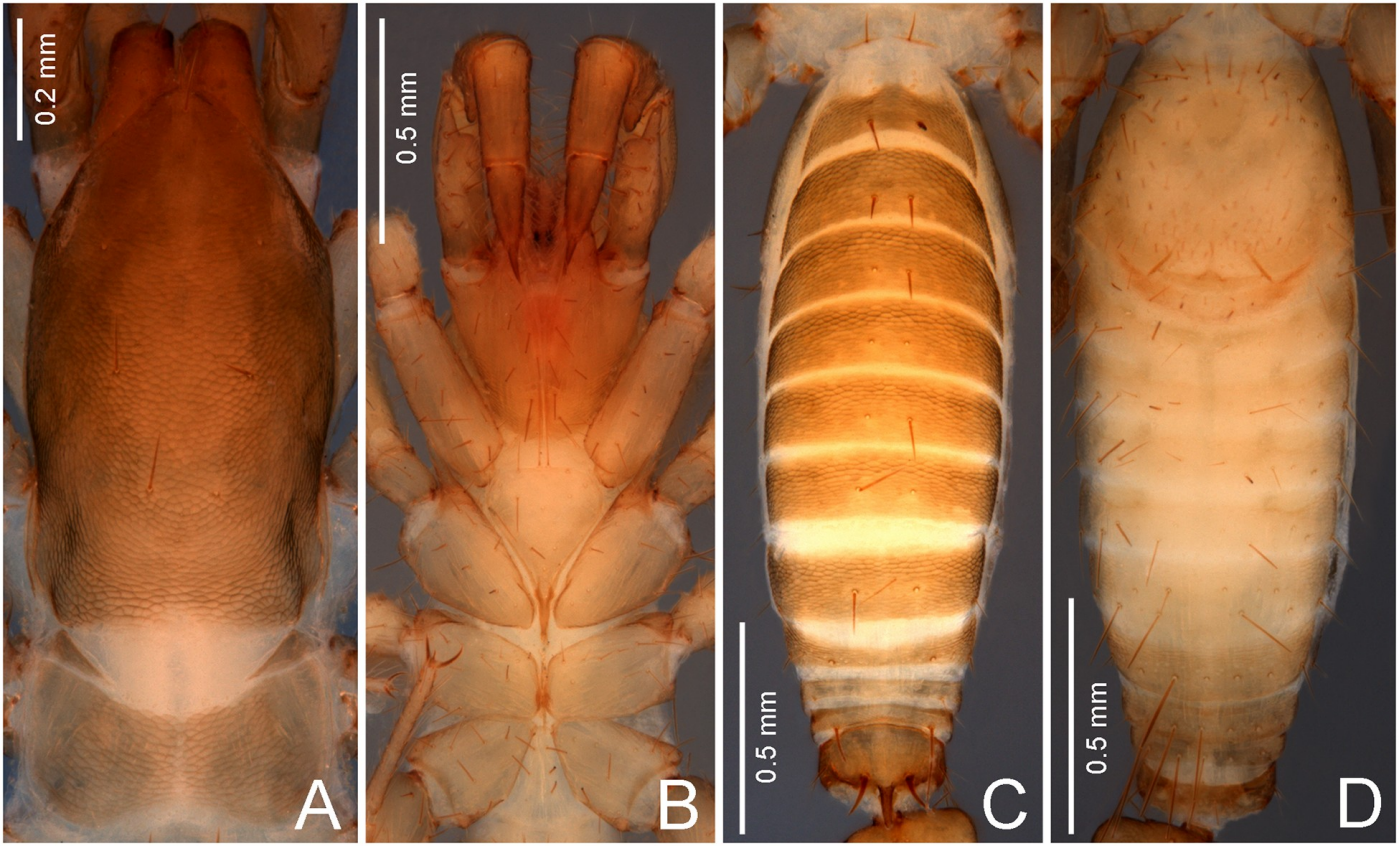
Details of the male holotype of *Surazomus saturninoae* sp. nov. (A) Peltidia, dorsal view. (B) Prosomal sterna, leg coxae and pedipalps, ventral view. (C-D) Abdomen: (C) dorsal view, (D) ventral view.

**Fig 3:**
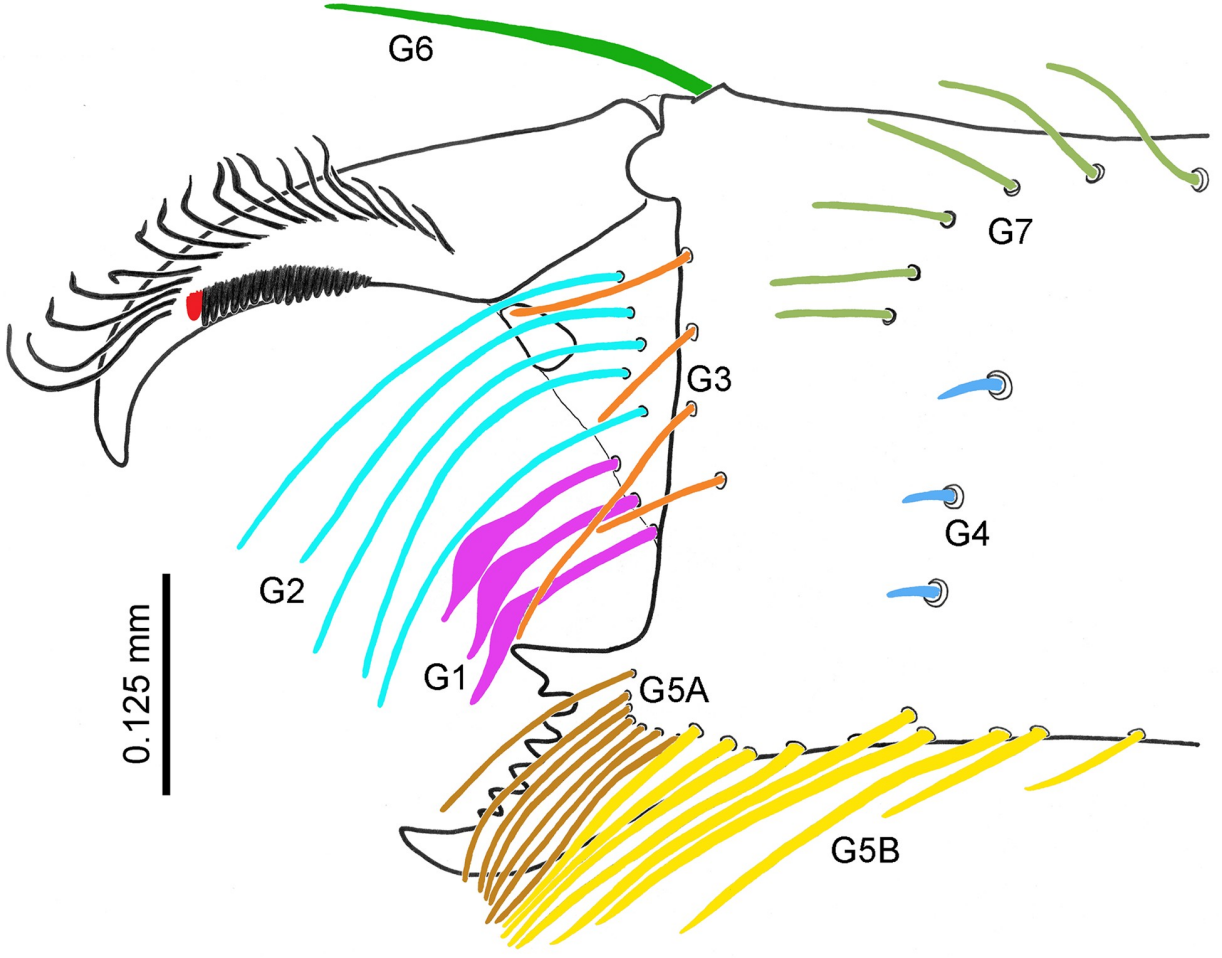
Chelicera setation of *Surazomus saturninoae* sp. nov. Male holotype, mesal view (guard tooth in red).

**Diagnosis**. Species of the *arboreus*-group of *Surazomus* may be recognized by the males having pentagonal flagellum and bearing only two dorsal coupling pockets: one in front of Dm1 (AP), and another between Dm1 and Dm4 (PP), and by having a single large posterodorsal process on tergite XII (see Cokendolpher & Reddell [23]: figs 11, 13, 20 and 23; Bonaldo & Pinto-da-Rocha [24]: figs 2 and 3). Also, females have two pairs of slender, elongated spermathecal lobes with small bulbs followed by terminal constrictions (see Cokendolpher & Reddell [23]: figs 14, 15, 24 and 25).

#### *Surazomus saturninoae* sp. nov.

urn:lsid:zoobank.org:act:D3953D2A-0898-4F24-933B-089D2CA3BF80 (Figs 1–6, 7F, 7G, 8E, 8F and 9D).

**Fig 4:**
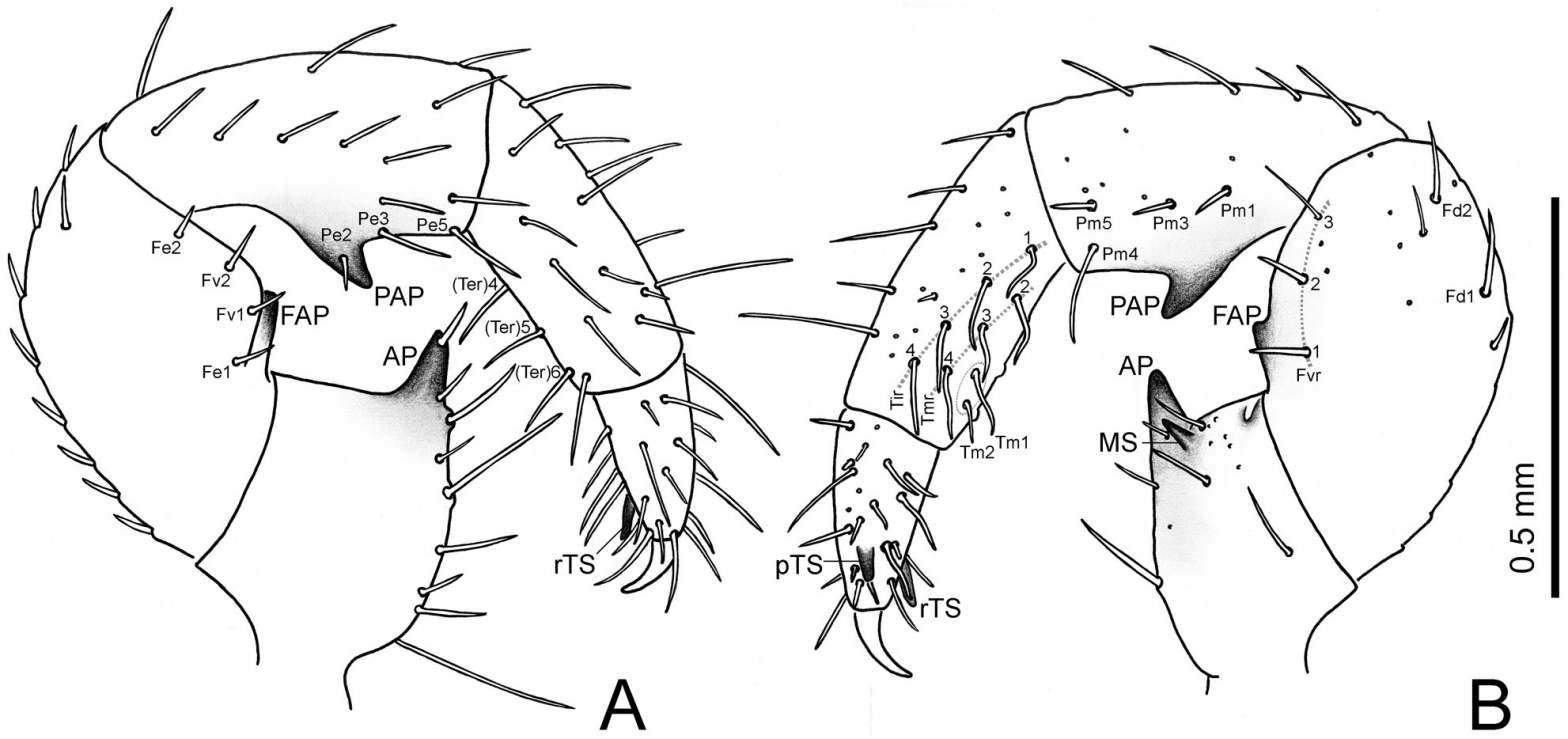
Right pedipalp of *Surazomus saturninoae* sp. nov. Male holotype: (A) retrolateral view, (B) prolateral view.

**Fig 5:**
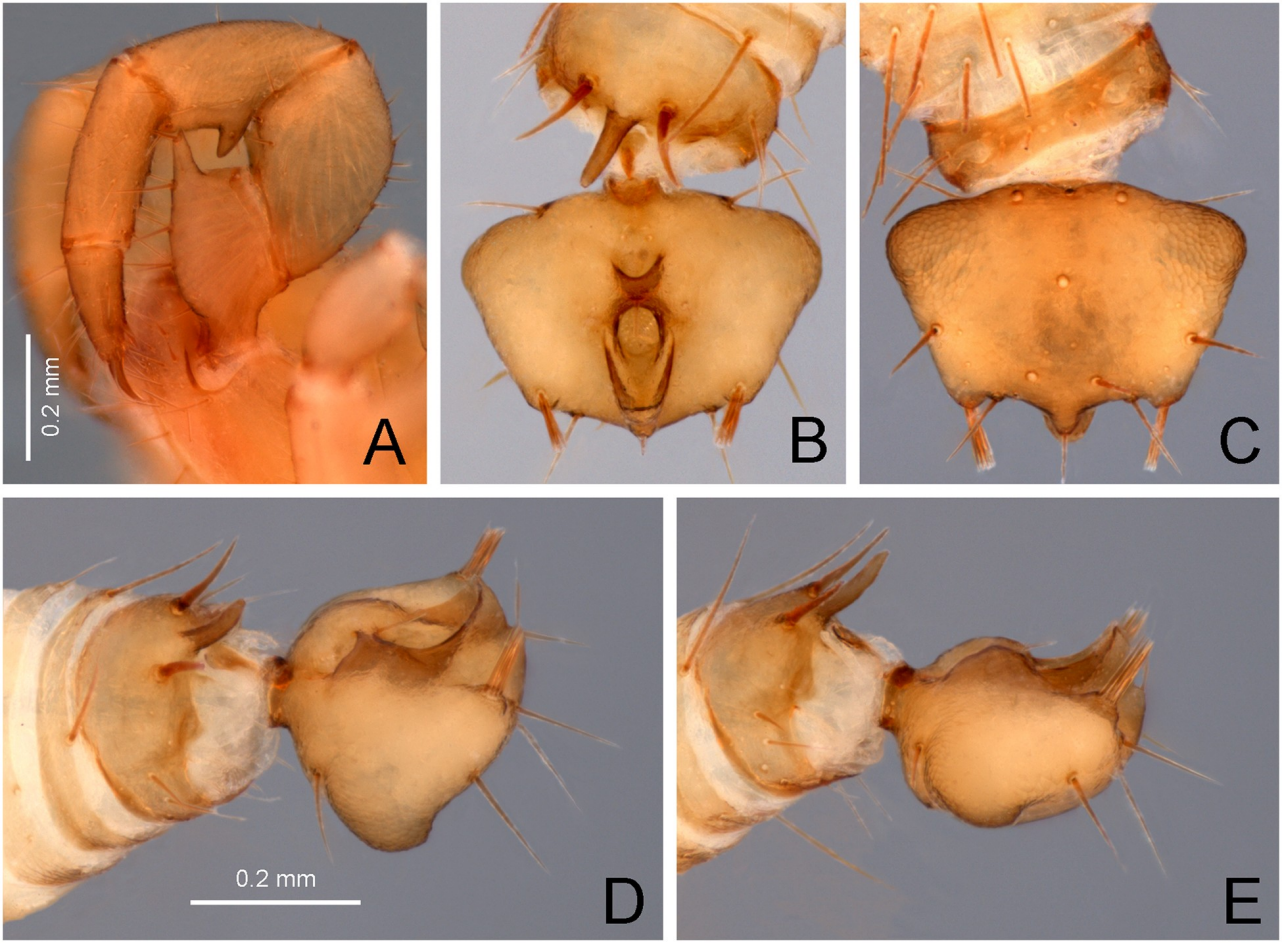
Pedipalp and flagellum of the male holotype of *Surazomus saturninoae* sp. nov. (A) Detail of left pedipalp, retrolateral view. (B-E) Flagellum: (B) dorsal view, (C) ventral view, (D) dorsolateral view, (E) and lateral view.

**Fig 6:**
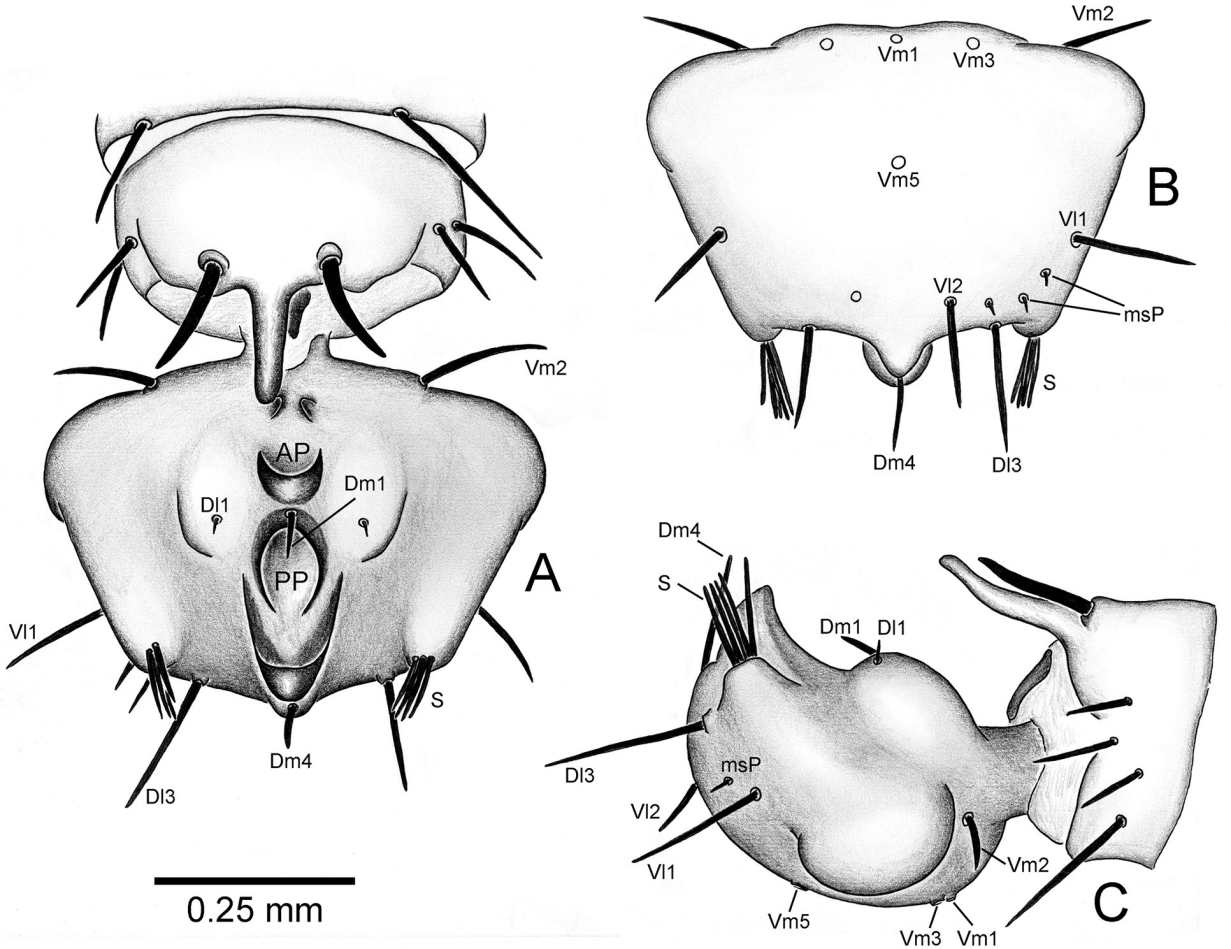
Flagellar setae of male holotype of *Surazomus saturninoae* sp. nov. (A) Dorsal view. (B) Ventral view. (C) Lateral view.

**Fig 7:**
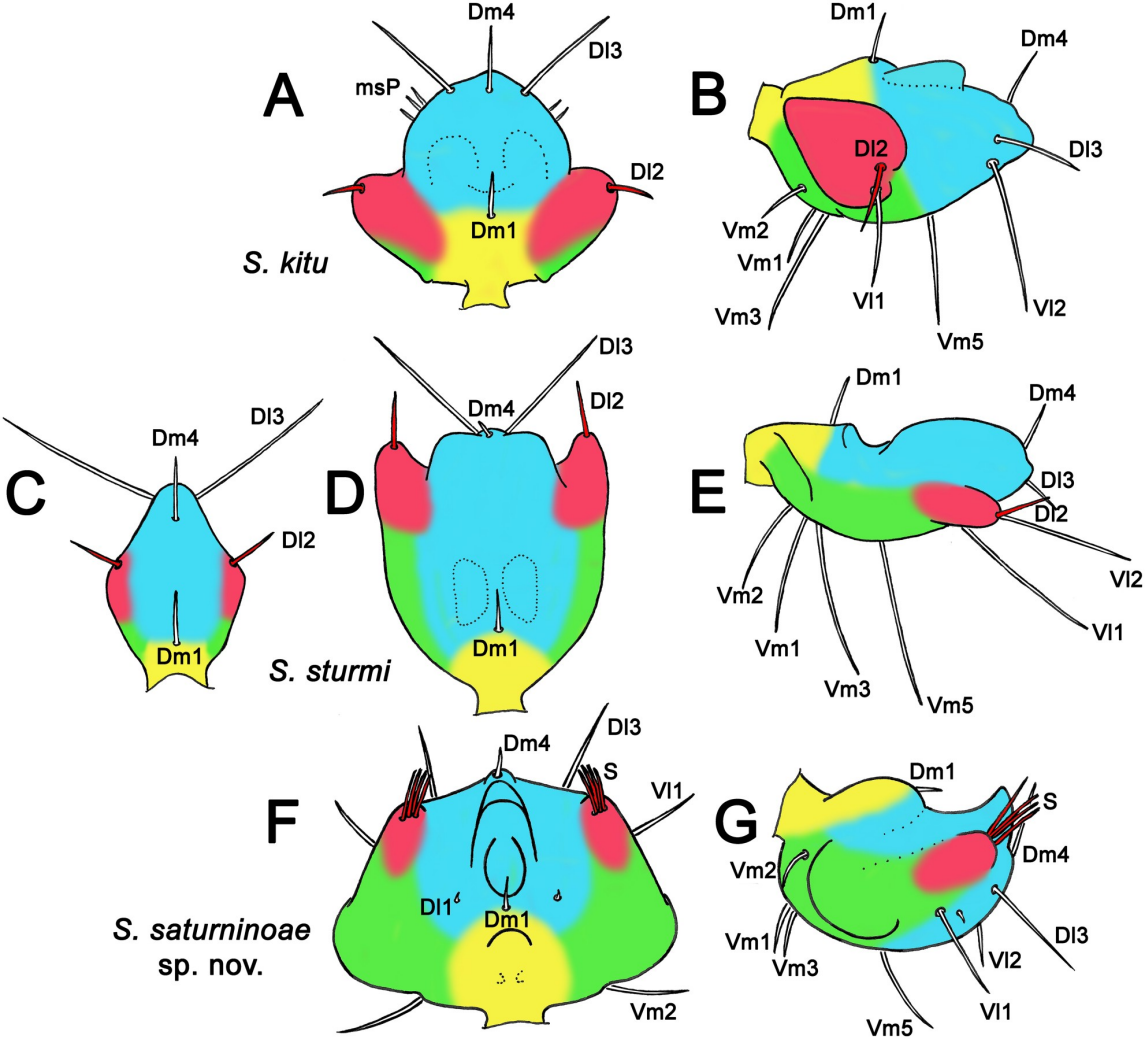
Proposal of homologies in male flagellar body and setae of *Surazomus* spp. Different colors show homologous portions. (A-B) Adult male of *S*. *kitu*: (A) dorsal view, (B) lateral view. (C-E) *S*. *sturmi*: (C) juvenile, dorsal view, (D) adult male, dorsal view, (E) adult male, lateral view. (F-G) Adult male of *S*. *saturninoae* **sp. nov**.: (F) dorsal view, (G) lateral view. Redrawing from: A-B: Villarreal *et al*. ([13]: figs 4A and 4C); C: Kraus ([22]: fig 9); D: Armas ([3]: fig 18A); E: Kraus ([22]: fig 8), with seta modifications to match the photograph given by Sturm ([7]: fig 22A). Yellow shows a small portion that includes the stalk and extends dorsally to the base of Dm1. Blue shows a median region that extends from the base of Dm1 to the base of Vm5 and includes the tip of the flagellum. Red areas show the lateral lobes, on which the Dl2 (also marked in red) are attached. Green areas connect the yellow stalk to the red lobes and are more developed ventrally, extending to the base of Vm5.

**Fig 8:**
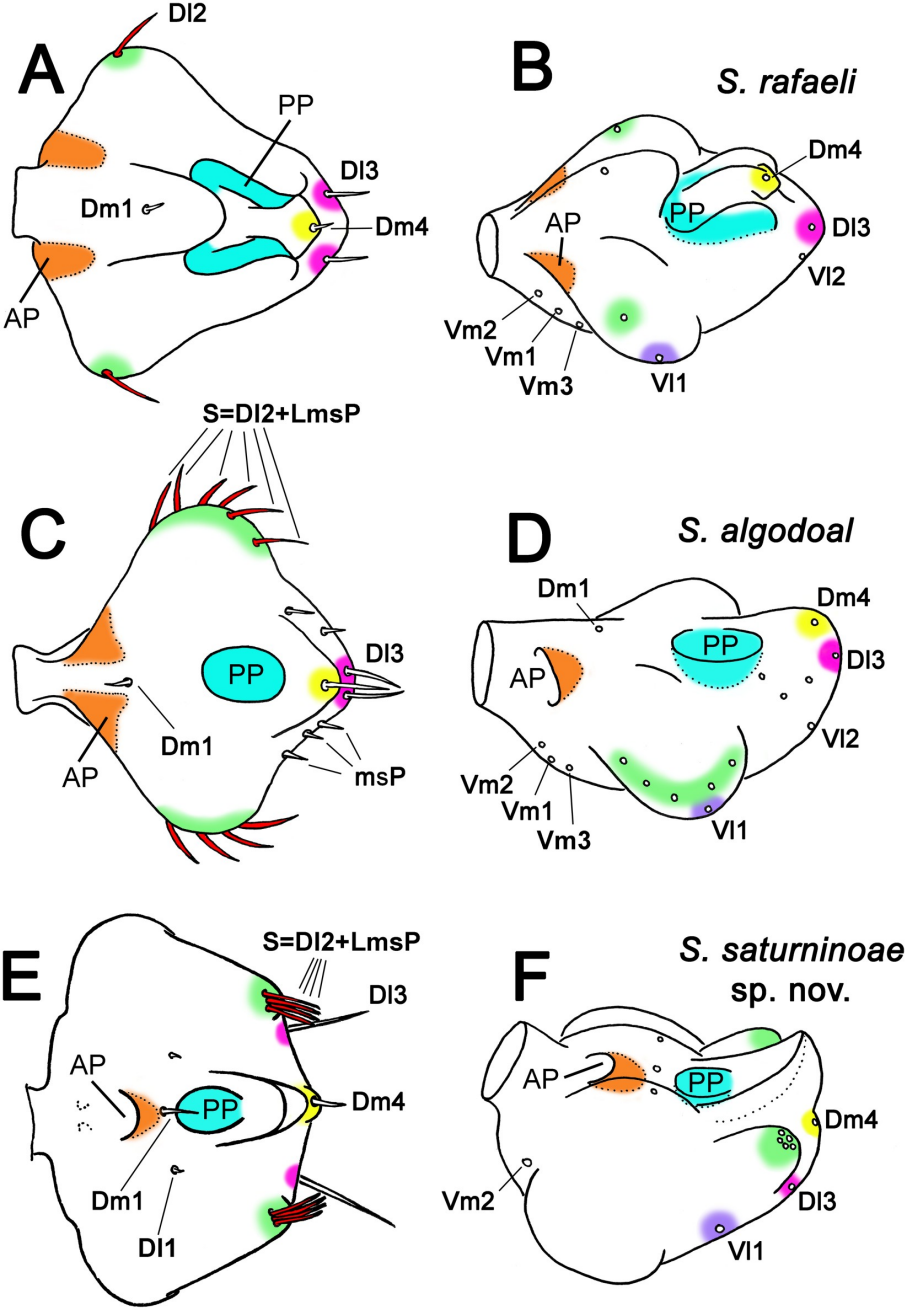
Proposal of homologies in male flagellar setae and coupling pockets of *Surazomus* spp. Different colors show homologies. (A-B) *Surazomus rafaeli*: (A) dorsal view, (B) dorsolateral view. (C-D) *Surazomus algodoal*: (C) dorsal view, (D) dorsolateral view. (E-F) *Surazomus saturninoae* **sp. nov**.: (E) dorsal view, (F) dorsolateral view. Redrawing from: A: Salvatierra [18]: fig 10; C: Ruiz & Valente [17]: fig 11; B, D, F: free-hand reconstructions based on dorsal views and on Salvatierra ([18]: fig 8) and Ruiz & Valente ([17]: fig 13). Abbreviations: AP: anterior pocket; PP: posterior pocket.

**Fig 9:**
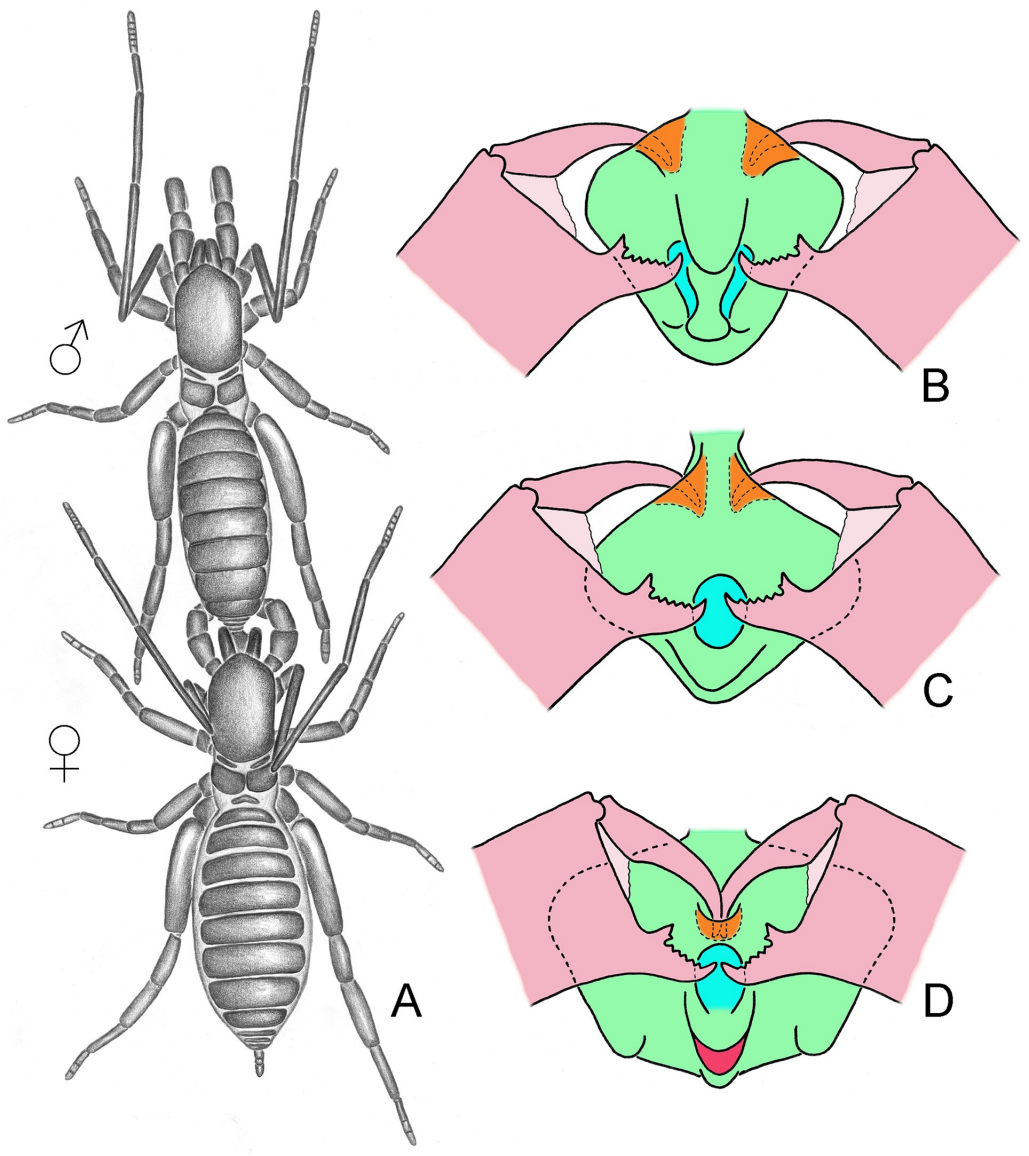
Mating march in *Surazomus*. (A) Male dragging locked female by the flagellum (note female chelicerae in vertical position). (B) Hypothesis of the anchoring mechanism for the species with paired AP and paired PP (four supporting points). (C) Hypothesis of the anchoring mechanism for *S*. *algodoal* (three supporting points); (D) Hypothesis of the anchoring mechanism for the species of the *arboreus*-group (two supporting points). Color explanation: (green) male flagellum; (pink) female chelicerae (illustrated in lateral view for better visualization of the hypotheses of anchoring machanism); (orange) AP; (blue) PP; (red) hood.

**Type material**. Holotype: male from Comunidade São Sebastião (02°05’07.5”S, 50°29’36.9”W), Igarapé Sequitã, Bagre, Pará, Brazil, 27.VI-02.VII.2016, R. Saturnino *et al*., pitfall trap in rain forest (MPEG SCZ 0039).

**Diagnosis from other species of the *arboreus*-group**. Male: the overall shape of the flagellum of *S*. *saturninoae* sp. nov. is similar to that of *S*. *arboreus*, but the flagellum is as wide as long in *S*. *arboreus* (see Cokendolpher & Reddell [23]: figs 11–13), while it is wider than long in *S*. *saturninoae* (Fig 6A). Also, the posterior dorsal hood (or dorso-median eminence) of *S*. *arboreus* is far from the posterior border of the flagellum, while it is close to the border in *S*. *saturninoae* (Fig 9D, in red). The widest portion of the flagellum in *S*. *paitit* is close to its middle length (see Bonaldo & Pinto-da-Rocha [24]: figs 2–4), while the widest portion of the flagellum in *S*. *saturninoae* is near its anterior border (Fig 6A). The posterodorsal process of abdominal segment XII of *S*. *manaus* (see Cokendolpher & Reddell [23]: figs 20–23) and *S*. *saturninoae* (Fig 6A and 6C) is long, but the flagellum of *S*. *manaus* has parallel sides with the posterior lobes much longer than the flagellum body, while the flagellum in *S*. *saturninoae* has oblique sides and shorter posterior lobes. Female is unknown.

**Etymology**. The specific epithet honors our friend, arachnologist Dr Regiane Saturnino, who collected the holotype. Noun in genitive case.

**Description. Male holotype**. Total length: 3.0 (not including the flagellum). Integument finely reticulated (Fig 2A and 2C). Coloration (Fig 1): sclerotized areas light brown, except for the reddish-brown chelicerae and proximally and distally pedipalps. Patella I totally pigmented. Membranous areas pale. Body setae reddish brown.

**Prosoma** (Figs 1, 2A and 2B). Length: 1.23. Propeltidium: 0.88 long, with 2 setae on anterior process (one behind the other); 3 pairs of dorsal setae; white eyespot elongate. Metapeltidium divided by a narrow white line. Anterior sternum only with one transverse line of five setae near the anterior border. Posterior sternum with three pairs of setae.

**Chelicerae** (Fig 3). Movable finger with guard tooth (Fig 3, red, behind serrula), serrula with 17 hyaline teeth and a row of 18 long setae. Fixed finger with a bifid basal tooth, followed by five small teeth and a larger, recurved tooth, with an acute apex. Setation: G1 with 3 spatulate setae, with spicules on distal half of peduncles; G2 with 5 subequal setae, almost as long as movable finger; G3 with 4 setae; G4 with 3 setae; G5 (A) with 7 similar-sized setae, almost as long as fixed finger; G5 (B) with 10 setae: seven along the ventral edge and three arising more mesally (ectal setae not counted); G6 with one smooth seta, longer than half the length of the movable finger; G7 with 6 setae. Setal group formula: 3-5-4-3-(7+10)-1-6.

**Pedipalp** (Figs 4A, 4B and 5A). Stout. Trochanter: with ten ventral setae, and a triangular, acute apical process (AP), bearing distally another seta; prolaterally with an acute mesal spur (MS), four additional setae and small tubercles. Femur: subglobose, 1.7 times longer than high; dorsal edge with a row of eight setae and over 4 times longer than ventral edge; retrolaterally with Fe1, Fv1, Fv2, Fe2 and one additional, subdistal, subdorsal seta; prolaterally with Fvr with setae 1, 2 and 3, and with Fd1, Fd2 and two additional setae, surrounded by small pores; ventrally with a developed femoral apophysis (FAP). Patella: cylindrical, straight, 2.3 longer than high, with a large patellar apophysis (PAP); dorsally with seven setae; prolaterally with Pe2 (on PAP), Pe3 and Pe5, and eight other unnamed setae; retrolaterally with Pm1, Pm3, Pm4 and Pm5, surrounded by small pores. Tibia: cylindrical, straight, 2.7 times longer than high; Ter with setae 4, 5 and 6; Tir with setae 1, 2, 3 and 4; Tmr with setae 2, 3 and 4; two other Tm setae; additionally, there are several other dorsal and retrolateral setae and small pores. Tarsus: conical, with asymmetrical spurs (prolateral and retrolateral tarsal spurs: pTS/rTS), and several setae and pores. Tarsal claw sharp and curved, half the tarsus length.

**Legs** [length of femur/ patella/ tibia/ (basitarsus + telotarsus)]. I 0.84/ 0.94/ 0.65/ (0.29+0.43); II 0.58/ 0.32/ 0.39/ (0.31+0.34); III 0.53/ 0.27/ 0.26/ (0.28+0.28); IV 0.86 (0.38 times longer than high)/ 0.32/ 0.55/ (0.48+0.36).

**Opisthosoma** (Figs 1, 2C and 2D). Tergite I poorly sclerotized. Tergite XII with a long, acute posterodorsal process. Dorsal setation: segments I-VII each with large pair of Dm only; VIII with a pair of Dm and a pair of Dl2; IX with a pair of Dm widely separate, a pair of smaller Dl1 and a pair of Dl2; X with pairs of Dl1 and Dl2; XI only with a pair of Dm widely separate; XII with a pair of well-developed Dm, and pairs of small Dl1 and Dl2. Sternite setation: II (genital plate; see Schultz [25] for a clear delimitation of abdominal segments) with an arch of large setae around the anterior and lateral borders, and many small setae scattered on the genital plate; III with pairs of Vm2, Vl1 and Vl2, five AS between the Vm2 pair, and two pairs of AS between Vm2 and Vl1; IV with pairs of Vm2, Vl1 and Vl2, a pair of AS between the Vm2 pair, and another pair of AS between Vm2 and Vl1; V with pairs of Vm2, Vl1 and Vl2, a pair of AS between the Vm2 pair, and two pairs of AS between Vm2 and Vl1; VI as IV; VII with pairs of Vm2, Vl1 and Vl2, and a pair of AS between Vm2 and Vl1; VIII as IV; IX-X with a median Vm1, and pairs of Vm2, Vl1 and Vl2; XI with a median Vm1, and pairs of Vm2 and Vl1; XII with pairs of Vm2, Vl1 and Vl2.

**Flagellum** (Figs 5B–5E and 6). 0.31 long, 0.41 wide, pentagonal, widest near anterior border; posteriorly with a pair of lobes; dorsally with a small anterior pair of pores, and with an anterior pocket (AP) facing posterodorsal process of segment XII; a second, median, posterior pocket (PP) faces posteriorly and is combined with a posterior hood (Fig 9D, in red). Dorsal setation (see Discussion about setal homologies below): centralized Dm1 between the dorsal pockets; Dl1 microsetae on each side of Dm1; Dm4 distal, behind the posterior hood; a brush (S) of five long setae, placed on the posterior lobes; Dl3 pair distally. Ventral setation: median Vm1 and a pair of Vm2 near the stalk, immediately followed by a pair of Vm3 (Vm2 widely separated, visible in dorsal view); Vm5 centralized; a pair of Vl1 on the lateral border, and a pair of Vl2 more distally. Additionally, the flagellum has about three distal msP on each side, ventrally between Vl1 and Vl2.

**Female**. Unknown.

**Distribution**. Known only from type locality.

**Natural history**. The single specimen was collected with pitfall trap in primary upland Amazonian Rain forest (Terra Firme) from Bagre, municipality in eastern Amazon, state of Pará, Brazil. *Surazomus saturninoae* **sp. nov**. is the third species of the genus collected from eastern Amazon. The two others were also collected from state of Pará, Brazil, *S*. *paitit* from the upland Amazonian Rain forest of Caxiuanã, and *S*. *algodoal* from the dry forest (Restinga) of Algodoal Island.

### Synopsis and comments on chaetotaxy in hubbardiines

Based on a diversity of Hubbardiinae from Australia, Harvey [11] proposed the first attempt to keep track of the larger flagellar setae. Cokendolpher & Reddell [9] used the initial proposal by Harvey [11] to map the setae of the flagellum of Protoschizomidae. They gave also attention to the smaller flagellar setae and, in order to keep setal homology between the two families, renumbered them in Hubbardiinae. Further reanalyses of flagellar setae in Hubbardiinae were made by Villarreal *et al*. [12,13] and Moreno-González *et al*. [14]. Monjaraz-Ruedas *et al*. [15] compared the previous descriptions (Hubbardiinae and Protoschizomidae) with Megaschizominae and tested the homologies of the female flagellomeres and setae across all Schizomida in a phylogenetic analysis, which resulted in newer changes, including homologies in male flagella. For a compilation of knowledge on flagellar setae in Hubbardiinae through time, see Table 1.

**Table 1:** Compilation of knowledge on flagellar setae in Hubbardiinae through time. Modified from Moreno-González *et al*. [14] to show homologies and the newer proposals. Notation: (-) = not found or unnamed; (→) = renumbering due to new findings. Uppercase/lowercase for the first letter is ignored to ease comparisons.

	Harvey [11]: based only on large setae of hubbardiines		Cokendolpher & Reddell [9]: renumbered the setae of hubbardiines proposed by Harvey [11] for homology with protoschizomids; included protoschizomid microsetae		Villarreal *et al*. [12]: found Dm3 microsetae in male hubbardiines		Moreno-González *et al*. [14] found Dm3 and Dl4 in female, and Dl1 in male hubbardiines (old Dl1 renumberd as Dl2)		Monjaraz-Ruedas *et al*. [15]: reevaluated and tested homologies of female flagellomeres and setae in Schizomida (Teruel [26] had already informally renumbered the old Dl1 as Dl2 in females)	
Setae	Male	Female	Male	Female	Male	Female	Male	Female	Male	Female
Single	Dm1	Dm1	Dm1	Dm1	Dm1	Dm1	Dm1	Dm1	Dm1	Dm1
	Dm2	Dm2	Dm2→Dm4	Dm2→Dm4	Dm4	Dm4	Dm4	Dm4	Dm4	Dm4
	Vm1	Vm1	Vm1	Vm1	Vm1	Vm1	Vm1	Vm1	Vm1	Vm1
	Vm4	Vm4	Vm4→Vm5	Vm4→Vm5	Vm5	Vm5	Vm5	Vm5	Vm5	Vm5
Paired	-	-	-	-	Dm3	-	Dm3*	Dm3	Dm3→Dl1	Dm3→Dl1
	-	-	-	-	-	-	Dl1	-	**	-
	Dl1	Dl1	Dl1	Dl1	Dl1	Dl1	Dl1→Dl2	Dl1	Dl2	Dl1→Dl2
	Dl2	Dl2	Dl2→Dl3	Dl2→Dl3	Dl3	Dl3	Dl3	Dl3	Dl3	Dl3
	-	-	-	-	-	-	-	Dl4	-	Dl4
	Vl1	Vl1	Vl1	Vl1	Vl1	Vl1	Vl1	Vl1	Vl1	Vl1
	Vl2	Vl2	Vl2	Vl2	Vl2	Vl2	Vl2	Vl2	Vl2	Vl2
	Vm2	Vm2	Vm2	Vm2	Vm2	Vm2	Vm2	Vm2	Vm2	Vm2
	Vm3	Vm3	Vm3→Vm4	Vm3→Vm4	Vm4	Vm4	Vm4	Vm4	Vm4→Vm3	Vm4→Vm3

* Villarreal *et al*. [13] found another pair of Dm3 microsetae. This second pair was referred as Dm3B and the original Dm3 as Dm3A.

** Monjaraz-Ruedas *et al*. [15] ignored the “Dl1” pair of setae found by Moreno-González *et al*. [14]; Moreno-González & Villarreal [16] drew again attention to male flagellar microsetae, including a “lateral (pair) over the pedicel”, their old “Dl1”, which they treated as NA.

Monjaraz-Ruedas *et al*. [15] stated that in Hubbardiinae the flagellomere IV was lost and flagellomeres V and VI were fused in the ancestor. Hence, only flagellomeres I, II, III, V+VI are present in Hubbardiinae, a pattern of four flagellomeres seen in several genera. However, in other genera of Hubbardiinae, flagellomere III is also fused with flagellomere V+VI, resulting in a female flagellum with only three flagellomeres (I, II, III+V+VI), as observed in *Surazomus*, for instance.

In hubbardiine females, flagellomere I bears no seta. In flagellomere II, there is a median Dm1, a median Vm1 and a pair of Vm2. When flagellomere III is free, there is a pair of Dl1 and a pair of Vm3. In flagellomere V+VI there is a median Dm4, and a median Vm5, and pairs of Vl1, Vl2, Dl2, Dl3 and Dl4. Setae of flagellomeres III and V+VI may be all on the same flagellomere in species with flagellomere III fused with V+VI [15]. Studying high-quality original descriptions, we observe that, even in species with only three flagellomeres, setae of the distal flagellomere cluster at different portions, revealing their origin prior to the fusion: Dl1 is usually aligned with Vm3 (originally from flagellomere III, as in Megaschizominae); Dl2 is usually aligned with Vl1, and close to Dm4 and Vm5 (all originally from flagellomere V, as in Megaschizominae); Dl3 is aligned with Vl2, and close to Dl4 (all originally from flagellomere VI, as in Megaschizominae).

In male flagella of hubbardiines, although being composed of a single flagellomere, different setae also cluster along the flagellum, similar to what happens in females. In most species, Dm1 is aligned at the base of the flagellum with the W-pattern formed by Vm1, Vm2 and Vm3 pairs. Generally, at the middle of the male flagellum and close to its wider area, Vm5 forms a cluster with Dl2 and Vl1 (Dl2 aligned with Vl1). Distally Dm4 forms a cluster with Dl3 and Vl2 (Dl3 aligned with Vl2). This pattern of setal distribution along the flagellum is easily understood in species with long flagella, such as *Piaroa* spp, and in most species of *Surazomus* with trilobed flagella (see Moreno-González *et al*. [14]: figs 6 and 7; Villarreal *et al*. [13]: figs 6A and 6B).

### New homologies for male flagellar setae of *S*. *algodoal* and species of the *arboreus*-group, and flagellum lobe-folding within *Surazomus* (Figs 7 and 8)

Considering the discussion above, flagellar Dl2 seta is aligned with Vl1 in hubbardiines. Males of *Surazomus* with trilobed flagellum traditionally bear Dl2 aligned with Vl1 on the lateral lobes (Figs 7A, 7B, 8A and 8B). Some species have also pairs of microsetae laterally on the median lobe near Dm4, Dl3 and Vl2, known as “msP” [13]. Although having these “msP” on the median lobe, lateral lobes of the male flagellum of *S*. *algodoal* have the Vl1 pair and at least four pairs of additional dorsolateral setae (Fig 8C), also labeled as “msP” in the original description [17]. These additional setae present on the lateral lobes are not true “msP”, and a new hypothesis needs to be created to treat them. It is possible that the most proximal pair of this brush (S) of lobular “msP” may be homologous to the Dl2 pair, while the rest of them may be additional, enlarged microsetae on the lobes (Lmsp = lobular microseta patch; Fig 8C and 8D). A similar phenomenon of enlargement of microsetae occurs in *Naderiore carajas* Pinto-da-Rocha, Andrade & Moreno-González, 2016, in which microsetae Dl1 (described as Dm3) became macrosetae (see Pinto-da-Rocha *et al*. [27]: fig 1). Several additional, enlarged setae are also present in the male flagellum of *Reddellzomus cubensis* Armas, 2002, which is covered with tens of macrosetae with no correspondence to setae of other groups (see Armas [28]: figs 3A and 3B). This hypothesis of additional, enlarged lobular microsetae in *S*. *algodoal* and their homology with the brush of the *arboreus*-group needs to be tested in future studies, especially if new, related species are found.

The male flagellum of species in the *arboreus*-group bears no dorsolateral setae on lateral margin. In these species, the lobes bearing multiple setae are on the posterior portion of the flagellum, but they are aligned with the Vl1 pair (Figs 7F, 7G, 8E and 8F). Based on that alignment, here we propose that the many pairs of setae forming a brush (S; originally proposed by Cokendolpher & Reddell [23] as a modification of Dl3) on the posterior lobes of the species of the *arboreus*-group (Fig 7F and 7G) may be instead homologous to the Dl2 and the several pairs of enlarged microsetae (Dl2+LmsP) seen on the lateral lobes of the male flagellum of *S*. *algodoal* (Fig 8C), differing only by the folding backwards of these lobes. Moreover, we wonder if the “micropores” seen on the posterior lobes of *S*. *manaus* [23] may be, in fact, the bases of the setae of the brush (S = Dl2+LmsP) that were rubbed off prior to the description.

The proposal of homology between the lateral lobes of the trilobed flagella and the posterior lobes of the pentagonal flagella (Fig 7) is also supported by the posterior position of Vl1 in these species, and additional observations in *S*. *sturmi*. Regardless the relationship of *S*. *sturmi* with the species with pentagonal flagella, in *S*. *sturmi* the juvenile male has a trilobed flagellum with the lateral lobes bearing the Dl2 pair (Fig 7C; see also Kraus [22]: fig 9), while adult males, however, have the lobes bearing the Dl2 pointing backwards (Fig 7D and 7E; see also Rowland & Reddell [29]: fig 42). This hypothesis of homology between the two types of lobes in *Surazomus* needs to be tested in future studies, especially if new species are found.

Also, in the species of the *arboreus*-group of *Surazomus*, the basal portion of the flagellum is widened. This conformation aligns all the five setae of the basal, ventral W-pattern (Vm1, Vm2, Vm3) in a transverse line, placing the Vm2 pair wide apart on the proximal border (Fig 6A–6C). This fact is the probable cause of the misleading identification of this pair as “Vl1” by Cokendolpher & Reddell [23] for *S*. *arboreus* and *S*. *manaus*, which was followed by Bonaldo & Pinto-da-Rocha [24] for *S*. *paitit*. According to this new interpretation, we homologized all the setae present in the descriptions of the species of the *arboreus*-group (Table 2), a hypothesis to be tested in future studies.

**Table 2:** Homology of flagellar setae among males of members of the *arboreus* goup of *Surazomus* and *S*. *algodoal*. Notation: (-) = absent; Abbreviations: (S) = setal brush; (msP) = microseta patch; (LmsP) = lobular microseta patch.

*S*. *saturninoae* sp. nov.	*S*. *algodoal* [17]	*S*. *arboreus* [23]	*S*. *manaus* [23]	*S*. *paitit* [24]
Dm1	Dm1	dm1	dm1	dm1
Dl1	*	**	-	-
Dm4	Dm4	dm4	dm4	dm4
S = Dl2+LmsP	msP	s = dl3	***	dl3
distal msP	msP	-	-	-
Dl3	Dl3	vl2	vl2	vl2
Vm1	Vm1	vm1	vm1	vm1
Vm2	Vm2	vl1	vl1	vl1
Vm3	Vm4	vm2	vm2	vm2
Vm5	Vm5	vm5	vm5	vm5
Vl1	Vl1	dl1	dl1	dl1
Vl2	Vl2	vm4	vm4	vm4

* ignored in the original description [17], but seen in Ruiz & Valente [17]: figs 9 and 10.

** originally ignored [23], but present, according to Villarreal *et al*. [12].

*** originally considered absent [23], herein homologized with the “micropores” on posterior lobes.

### Homologies of the coupling pockets within *Surazomus* (Fig 8), and hypotheses for the female anchorage onto male flagellum (Fig 9)

Most species of *Surazomus* have a pair of elongate dorsal depressions on the posterior portion of the male flagellum, treated herein as “posterior pockets” (PP). This is commonly seen in species with trilobed flagellum, such as *S*. *rafaeli* (Fig 8A and 8B). These elongate PP are almost parallel, but at an angle that aims them to the two sides of the flagellar stalk. The elongate PP are aligned with ventral depressions on the anterior border of the flagellum near the stalk, treated herein as “anterior pockets” (AP) (Fig 8A and 8B; see also Armas & Víquez [30]: fig 2A; Salvatierra [18]: figs 8 and 12). In *S*. *algodoal*, the PP are fused into a single median dorsal coupling pocket on the posterior half of the flagellum, instead of being a pair. Aligned to this median PP, this species has a pair of AP located more dorsally when compared to those of other species (Fig 8C and 8D; see also Ruiz & Valente [17]: figs 9 and 10). In the *arboreus*-group, the PP is similar to that of *S*. *algodoal*, nevertheless there is only another dorsal pocket anteriorly on the flagellum, instead of a pair of lateral pockets (Figs 6A, 8E and 8F).

The pair of posterior pockets of the male flagellum (PP) is located dorsally between Dm1 and Dm4 in *Surazomus*. In the *arboreus*-group and in *S*. *algodoal* there is only a median pocket, but located exactly between Dm1 and Dm4. Due to the similar shape and same position, we conclude that the single, median PP of *S*. *algodoal* and of the *arboreus*-group of *Surazomus* may be homologous to the pair of PP seen in the other species of the genus. Besides having a single, median PP, *S*. *algodoal* and the *arboreus*-group seem to share the pattern S = Dl2+LmsP (see above), which suggests a close phylogenetic relationship to be tested in future studies.

The same event seems to have occurred with the anterior pockets (AP). The AP pair is ventrally located and separate by the stalk in hubbardiines, such as in *Naderiore* and *Cangazomus* [27] (figs 2, 3, 5 and 6), *Calima* [31] (figs 3b, 3c, 7b and 7c), *Mayazomus* [20] (figs 19, 31, 45, 60, 72, 90 and 107); [21] (figs 9F and 9G), *Rowlandius* [32] (figs 2B and 2D), *Piaroa* [14] (figs 3, 6, 25 and 28), and in most species of *Surazomus*, being always anterior to Dm1, as seen in *S*. *rafaeli* (Fig 8A and 8B). From that pattern, within *Surazomus*, the AP pair began moving dorsally, becoming closer together, as we see in *S*. *algodoal* (Fig 8C and 8D). We propose that, from a similar state, the AP kept moving dorsally and, eventually, fused medially, giving rise to the sole AP seen in the *arboreus*-group, also anterior to Dm1, as in the other *Surazomus* (Figs 8E, 8F and 9D). The relocation and fusion of the AP dorsally push Dm1 (originally on flagellomere II in Schizomida) to the center of the flagellum, becoming transversely aligned with the Dl1 (originally on flagellomere III in Schizomida) (Fig 8E and 8F; compare with Villarreal *et al*. [12]: fig 15). Hence, we propose that the single, median AP of the species of the *arboreus*-group may be homologous to the AP pair present on the anterior border of the flagella of the other species of *Surazomus*, an explanation more parsimonious than the loss of the anterior pockets and the development of a new dorsal pocket. This hypothesis, however, needs to be tested in future studies.

The shape and position of the PP and AP on the male flagellum may be sexually selected. According to Sturm [6,7], the male schizomid flexes the flagellum 45° ventrally in front of the female, and the female holds it with the chelicerae. Then, the male flexes the flagellum back to its original position and locks the chelicerae of the female. The locked female is dragged forward, while the male lays the spermatophore on the ground. When the genital opening of the female is over the spermatophore, the male pushes her down to help sperm transfer.

Although the mating march was directly studied by Sturm [7] in 12 couples, he could not observe clearly the anchoring mechanism. Based on the proportions of the chelicerae and the male flagellum, and the position of the different pockets, we believe that, when the male flexes the flagellum 45° down, the female cheliceral teeth (fixed fingers) are placed inside the PP, while the fangs (movable fingers) hold the flagellum by the AP (Fig 9). The same mechanism seems to have been concluded by Sturm ([7]: 131; fig 22b), who described the PP as “paired depressions on the dorsal side”, in which “the ventral portion of the movable finger of the chelicera is most likely pressed onto” (in free translation). The AP were described by him as “chitin plates of the flagellum that serve as support for the chelicera claws” (in free translation). It seems that, during the mating march (Fig 9A), the chelicerae are held open at an angle in most species of *Surazomus*, which have paired PP, and paired AP on the sides of the stalk (Fig 9B), i.e., four supporting points. The long distance between the AP and the PP, combined with the dorsal and ventral position of these structures and the lateral flaps (or dorso-submedian eminences), seems to lock firmly the chelicerae of the female onto the male flagellum for the mating march. In the species with a single PP, the AP would progressively become close together and more dorsal (deviating from the stalk), until the complete fusion on the dorsum of the male flagellum in the *arboreus*-group. This new placement of the fused AP close to the fused PP would reduce the stability of the anchoring mechanism from four supporting points to only two, but this seems to have been overcome by the development of the dorsal hood (or dorso-median eminence). The dorsal hood right behind the PP (Fig 9D) would be pressed behind the cheliceral teeth and lock the parallel chelicerae of the female during the mating march. In *S*. *algodoal* (Fig 9C), the hood is not present (see also Ruiz & Valente [17]: figs 10 and 13), which could be the reason of the separate AP near the stalk, maximizing the stability of the anchoring with three supporting points.
